# The Myriapoda and Onychophora collection (MY) of the Muséum national d’Histoire naturelle (MNHN, Paris)

**DOI:** 10.3897/zookeys.518.10223

**Published:** 2015-08-25

**Authors:** Gwenaël Le Bras, Jean-Jacques Geoffroy, Laurent Albenga, Jean-Paul Mauriès

**Affiliations:** 1 Muséum national d’Histoire naturelle, Direction des collections, Unité de gestion des collections Arthropodes terrestres, CP 49/50, 57 rue Cuvier, 75231, Paris Cedex 05, France; 2 Muséum national d’Histoire naturelle, équipe e-ReColNAt, CP 49/50, 57 rue Cuvier, 75231, Paris Cedex 05, France; 3 Muséum national d’Histoire naturelle, Département Ecologie & Gestion de la Biodiversité, UMR 7204 CESCO CNRS-MNHN-UPMC, 4 avenue du Petit Château, 91800, Brunoy; 4 Attaché honoraire - Muséum national d’Histoire naturelle, Département Systématique & Evolution, CP 53, 57 rue Cuvier, 75231, Paris Cedex 05, France

**Keywords:** Occurrence, Specimen, Myriapoda, Chilopoda, Diplopoda, Pauropoda, Symphyla, Onychophora, Taxonomy, Specimens, Collections, Types

## Abstract

The Myriapoda and Onychophora collection dataset inventories the occurrence records of the collection of myriapods and onychophorans in the Muséum national d’Histoire naturelle, Paris. The dataset currently consists of 202 lots of onychophorans, representing all of those present, and almost ten thousand (9 795) lots of myriapods, representing 33 to 40% of the MNHN Myriapoda collection. This collection, which is of key historic importance, represents the results of two centuries of myriapod and onychophoran studies. The sources of the collection are worldwide, with a high representation for metropolitan France for the myriapods. None of the occurrences are yet georeferenced. Access to the dataset via the data portals of the MNHN and the GBIF has been made possible through the e-ReColNat project (ANR-11-INBS-0004).

The Myriapoda and Onychophora collection of MNHN is actively expanding, hence both the collection and dataset are in continuous growth. The dataset can be accessed through the portals of GBIF at http://www.gbif.org/dataset/3287044c-8c48-4ad6-81d4-4908071bc8db and the MNHN at http://science.mnhn.fr/institution/mnhn/collection/my/item/search/form.

## Description

Established in the second half of the 19th century, the MNHN’s combined collection of myriapods and onychophorans is still treated as one unit, mostly for historical and practical reasons.

The myriapod collection is a major resource for various studies on the group worldwide. The material comes from all around the world and represents the classes Pauropoda (2 extant orders), Symphyla, Chilopoda (5 extant orders) and Diplopoda (16 extant orders), collected from most terrestrial ecosystems. About 5 000 species of myriapods are represented in the collection, including a high number of historic samples. The myriapod collection comprises between 300 000 and 400 000 specimens. Nearly half of it constitutes the identified reference collection, the other half, which includes a potentially important number of taxa yet unknown to science, is awaiting study. The identified reference collection comprises between 25 000 and 30 000 lots, each containing from 1 to more than 300 specimens, stored in 4 639 jars. The identified reference collection includes between 3 000 and 3 500 lots, containing altogether 15 000 to 20 000 type specimens.

To date, the MNHN-MY dataset covers 9 795 lots of myriapods, consisting of 63 617 specimens, which represents between 33% and 40% of the reference collection.

The MNHN collection of onychophorans (commonly known as velvet worms) is one of the world’s most important for this group, including invaluable historical material, such as type specimens of species described by Louis Eugène Bouvier (1856–1944) in the early 1900s. Although only a small phylum, it is of key importance in several respects within the Metazoa, particularly regarding phylogenetic relationships between arthropods and other invertebrates (Oliveira et al. 2012). The collection boasts almost a hundred species, represented by 202 lots containing 279 specimens and 43 dissections. It is stored in 67 jars. The type material represents 56 lots containing 73 type specimens (plus 2 dissections of types).

This part of the collection is fully incorporated in the MNHN-MY dataset, which has since been used for studies on the group (Oliveira et al. 2012).

## History of the collection

**Myriapods**: The real creation of a myriapod collection dates back to the combining of the type specimens of Paul Gervais (1816–1879) and Pierre Hippolyte Lucas (1814–1899) in the 19th century. From 1890 to 1935, this collection was enriched by the donation of the material studied by Henry Wilfred Brolemann (1860–1933) [after World War I, Brolemann modified his legal name from Brölemann to Brolemann; hereafter we use the latter spelling]. This collection contains a very large number of samples, including many type specimens of taxa described by Brolemann himself, but also by many of the most famous myriapodologists of his time (e.g. Attems 1908). Thus the collection was enriched with material from metropolitan France related to Brolemann’s prolific work on the myriapod fauna of this sector, gathered by himself and by various collectors, institutes and programmes, such as Biospeleogica (Brolemann 1923, 1930, 1935, Duboscq et al. 1933; for a complete list of Brolemann’s publications, see the MyriaLit Database http://www.myriapodology.org/myrlit/). The collection was also enriched by samples collected during his many travels before he joined the MNHN (he lived in the United States of America and in Italy) (Duboscq 1933). Also, as a world authority on the group, Brolemann received material from all over the world for study (Brolemann 1896a, 1896b, 1897, 1898, 1909, 1920, 1922, 1926, MyriaLit Database). The MNHN collection had become a major centre for myriapodology attracting numerous international specialists who studied the collection and donate new material to the collection, such as Reginald Innes Pocock (1863–1947), Filippo Silvestri (1873–1949), Ralph Vary Chamberlin (1879–1967), Karl Wilhelm Verhoeff (1867–1945), Carl Attems (1868–1952), Otto Schubart (1900–1962) and Karl Kraepelin (1848–1915) (Kraepelin 1910a, 1910b). In addition, the collection received specimens from correspondents of the MNHN, such as Henri Gadeau de Kerville (1858–1940) (Anonymous 1941).

After 1940, the collections were enriched by donations to the Muséum national d’Histoire naturelle. The important collection built up by Paul Remy (1894–1962), who in particular studied the speleological fauna of Europe, the Balkans, northern America and northern Africa, greatly increased the MNHN-MY holdings of pauropods, a group that he studied extensively, as well as those of symphylans (Condé 1962, 1963). During the same period, the collection was enriched by the study collections of former students and collaborators of Brolemann, such as Jules Chalande (1854–1930) and Henri Ribaut (1872–1967) (Ribaut 1922). The subsequent growth and curation of the collection was the result of the work of Jean-Marie Demange, Jean-Paul Mauriès, Monique Nguyen Duy-Jacquemin and today Jean-Jacques Geoffroy and international specialists, such as Sergei Golovatch.

The collection continues to grow at a rate varying from several dozens to several thousands of specimens every year. Among the recent acquisitions, the most valuable for science are those from Madagascar, French Guiana, Brazil, different European ecosystems (high mountains, deep caves, transformed forests), China and south-east Asia, especially the material from the 2005 and 2006 expeditions to Clipperton Island and Santo (Vanuatu).

**Onychophorans**: The creation of the MNHN collection of onychophorans is closely linked with the interest that Bouvier developed for this group. Alongside his numerous interests and studies on crustaceans, pycnogonids, cetaceans and molluscs, Bouvier, was Director of Entomology at the MNHN from 1895 to 1931, and a pioneering researcher on onychophorans (Caulery 1944). He managed to obtain samples through institutional exchanges and described many new taxa from all over the distribution areas of the two extant onychophoran families (South America, southern Africa and Australasia). Based on this collection, Bouvier built his monograph of the onychophorans, which is a milestone for the scientific knowledge on this group (Bouvier 1905, 1907b). In 1907, when he published a catalogue of the collection, it already boasted 92 lots, containing 38 species plus about 6 varieties (Bouvier 1907a). Since Bouvier’s death in 1944, the collection has grown at a much slower rate.

In 2000, this collection was completely revised by one of the major specialists of this group, Hilke Ruhberg (University of Hamburg, Germany).

## Project details

**Project title**: Digitization and provision of the data from the Muséum national d’Histoire naturelle to the international community.

**Personnel**: Gwenaël Le Bras (data publisher, data manager), Jean-Jacques Geoffroy (curator, data manager, collection identifier, data collector), Laurent Albenga (data publisher, data manager), Jean-Paul Mauriès (data collector, collection identifier, data publisher, data manager, former curator).

**Funding**: e-ReColNAt : ANR-11-INBS-0004, Muséum national d’Histoire naturelle, Paris (MNHN).

**Study area descriptions/descriptor**: The dataset corresponds to those parts of the collection of myriapods and onychophorans of the MNHN that have already been entered into the database. The present dataset does not represent the totality of the collection, since its digitization is still incomplete. This collection is an invaluable legacy for knowledge of myriapods and onychophorans, in terms of its history, size and high proportion of type specimens. Uploading this dataset to shared database systems was therefore important for the future uses of the collection, to provide easy access by researchers and the general public to the detailed data it contains. This also provides better conservation of the data, because shared systems are better lasting solutions than local computers. The main goal of the e-ReColNAt project in this case was to refine and transfer the existing metadata from a local, mono-table database (4D system) to the MNHN’s shared collection database system (Oracle), and to allow its publishing through the MNHN web services and the GBIF portal. One entry corresponds to a lot, consisting of one to several specimens sharing the same collection data and belonging to the same taxon.

**Design description**: From the mid-1980s, the collection curators and researchers J.-P. Mauriès and J.-M. Demange have been digitizing the data, mostly in relation to their taxonomic work, on a local database (4D). By 2010, the database had reached ca. 10 000 entries. This database was originally created simply to be requested only by jar or lot identifier. Therefore, this large dataset needed both structuring and refining in order to allow multi-field requests before being integrated into the MNHN shared collection database system (Oracle) and connected to the GBIF infrastructure through an Integrated Publishing Toolkit (IPT) (Soberón et al. 2002). Initial work was conducted in 2011 by Laurent Albenga to upload all 202 entries for onychophorans. The remaining 9 795 myriapod entries have been refined in the scope of the e-ReColNAt project by Gwenaël Le Bras, and were committed to the MNHN shared collection database system on 27^th^ January 2015, being harvested by GBIF on 16^th^ March 2015. In addition to Oracle solutions and to the MNHN’s in-house JACIM systems for scientific management (http://collections.mnhn.fr/wiki/Wiki.jsp?page=Jacim_Documentation), OpenRefine (http://www.openrefine.org) proved to be a very precious tool in the refining process. Geographical analyses of the dataset, including the maps presented here, were made using Quantum GIS mapping software (http://www.qgis.org) and the Thematicmapping.org world borders dataset (http://thematicmapping.org/downloads/world_borders.php). The databasing is carried out at the lot level, with information concerning identification, nomenclatural status, number of specimens, sex and development state, geographical origin, type of collection, collecting method. The geographical coverage of the dataset reflects the activities of scientist’s with these collections during the past decades. The portions of the collection that have been recently studied tend to be more scattered (fewer specimens per lot and fewer lots per jar).

**Data published through GBIF**: http://collections.mnhn.fr/ipt/resource.do?r=mnhn-my

## Taxonomic coverage

**Figure 1.**
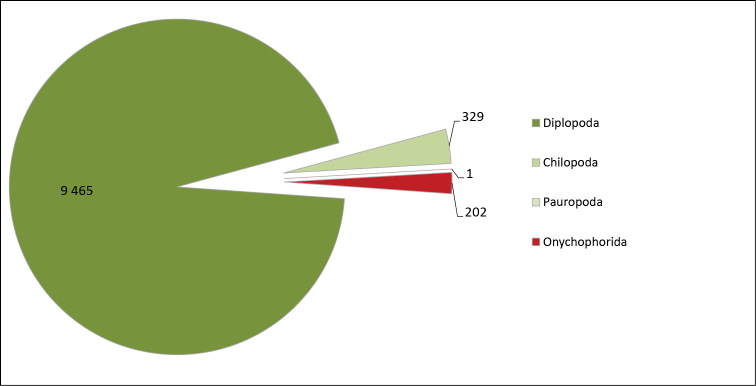
Taxonomic coverage (by class) of the MNHN-MY dataset in terms of number of lots. (Entries up to 27.01.2015)

**Figure 2.**
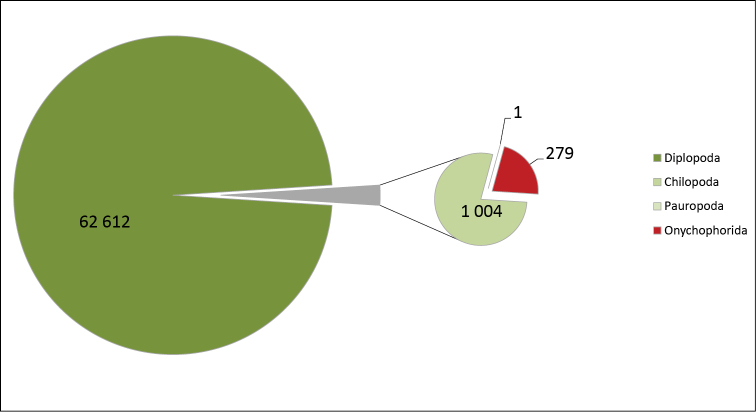
Taxonomic coverage (by class) of the MNHN-MY dataset in terms of number of specimens. (Entries up to 27.01.2015)

**Figure 3.**
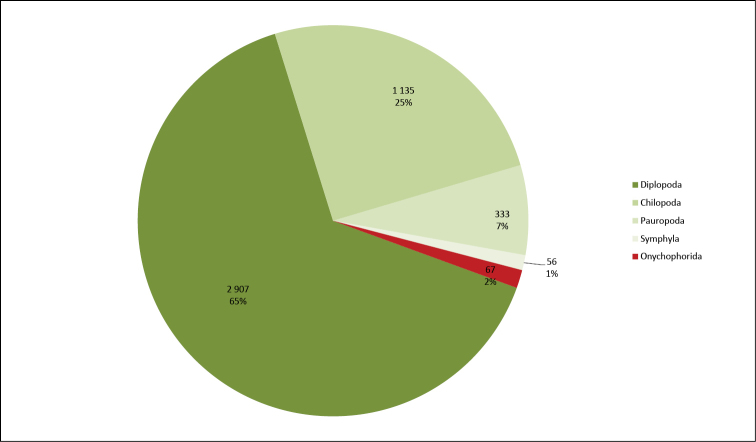
Taxonomic coverage (by classes) MNHN-MY reference collection in terms of number of jars.

**General taxonomic coverage description**: As depicted in Figures 1 and 2, to the exception of the onychophorans, the dataset reflects the scientific activities of the researchers carrying out the digitization. Consequently, even though the collection taxonomically covers all myriapods and onychophorans, the dataset mostly comprises millipedes. The digitization work was conducted together with a revision of the specimens concerned, and often leads to a splitting of the original lots into smaller ones. As a result of this, these two collections are also more scattered than the others, with less specimens per lot, as depicted in Figure 3. The taxonomy used in the dataset follows that used in the collection. Like that of most of the large historical collections around the world, it is in many ways outdated, and would be very difficult to update in practice (Soberón et al. 2002, Gaston and May 1992, Alroy 2002).

The onychophoran collection contains 48 species and 7 subspecies, belonging to 15 genera of the two currently known extant families.

## Taxonomic ranks

Kingdom: Animalia (animals)

Phylum: Arthropoda (arthropods)

Class: Chilopoda (centipedes)

Order: Geophilomorpha, Lithobiomorpha, Scolopendromorpha, Scutigeromorpha

Class: Diplopoda (millipedes)

Order: Callipodida, Chordeumatida, Glomerida, Glomeridesmida, Julida, Platydesmida, Polydesmida, Polyxenida, Polyzoniida, Siphonophorida, Sphaerotheriida, Spirobolida, Spirostreptida, Stemmiulida

Class: Pauropoda

Order: Tetramerocerata

Class: Symphyla

Phylum: Onychophora (velvet worms)

Class: Onychophorida

Order: Euonychophora

## Spatial coverage

**General spatial coverage**: No lots from this dataset have been georeferenced, but contemporary country names have been included when possible, according to ISO 3166 (http://www.iso.org/iso/country_codes.htm). The two main parts of the collection have a different spatial repartition, due in part to the distribution of the taxa represented, but also to a different history.

**Figure 4.**
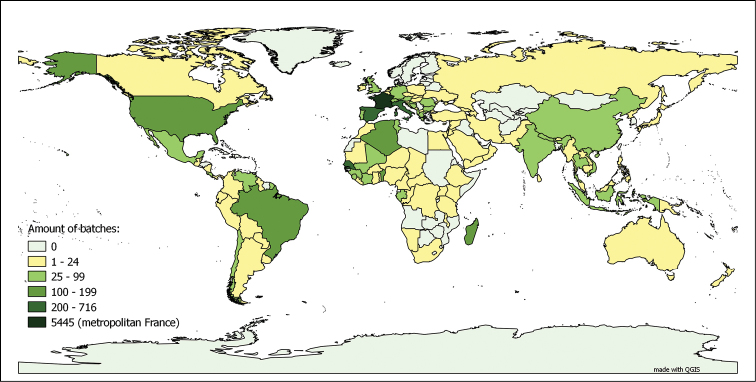
Origins of the myriapod lots in the MNHN-MY dataset. Based on the 9 587 lots bearing information on country of origin, out of 9 795 lots in the dataset (entries up to 27.01.2015)

**Figure 5.**
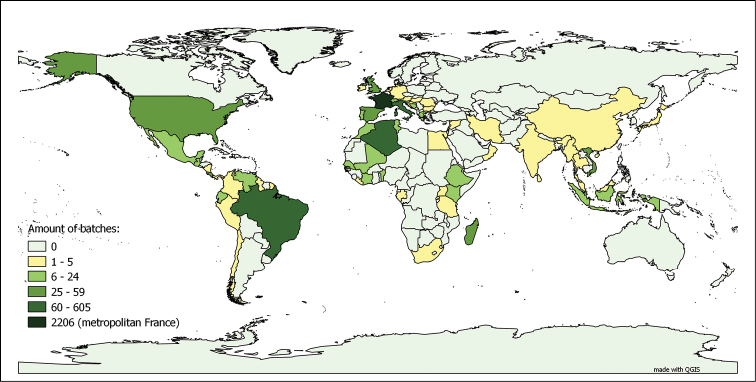
Origins of the myriapod lots collected prior to 1933 in the MNHN-MY dataset. Based on the 3 548 lots bearing information on country of origin, out of 3 559 lots collected before 1933 in the dataset (entries up to 27.01.2015)

**Myriapods**: The myriapod dataset is a collection for “metropolitan France and the world”. In fact, 56.80% of the databased lots were collected in metropolitan France, representing 72.64% of the total number of specimens in the collection. This is due to the fact that the number of specimens per lot collected in metropolitan France is significantly higher than the average number of specimens per lot for the rest of the world, including the French overseas territories. Even though the metropolitan France collection has been extensively studied (and consequently its lots have been split numerous times following their taxonomic revision), they are still, on average, larger than those coming from the rest of the world. The worldwide distribution, as depicted in Figure 4, is not homogeneous. Both characteristics of the dataset, as well as the taxonomic coverage (see above), reflect the main centres of interest of Demange’s and Mauriès’ research work. The influence of Brolemann’s studies on the faunas of Algeria, Brazil and the USA (Brolemann 1896b, 1897, 1909, 1931, MyriaLit http://www.myriapodology.org/myrlit/) is clearly visible in Figure 5.

**Figure 6.**
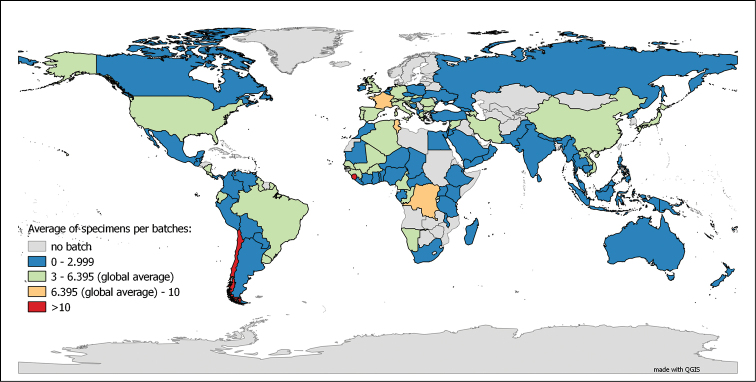
Average number of specimens per lot in the MNHN-MY dataset. (Entries up to 27.01.2015)

**Figure 7.**
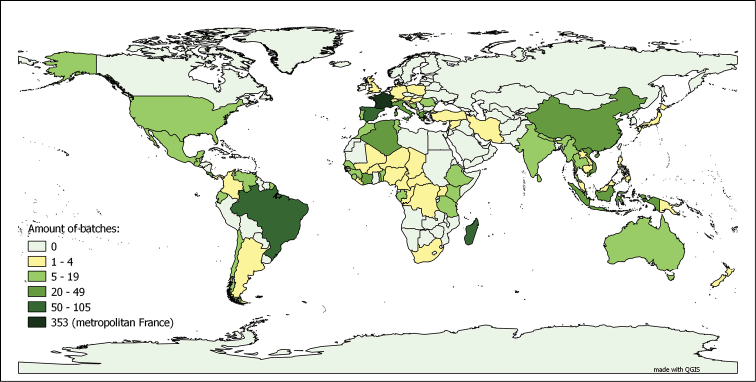
Origins of the myriapod lots containing type specimens in the MNHN-MY dataset. Based on the 1 137 lots of type specimens bearing information on country of origin, out of 1 170 lots containing types in the dataset (entries up to 27.01.2015)

It is, however, highly probable that the number of specimens from metropolitan France is over-represented in the dataset, compared to the whole collection. The same phenomenon is observed when interpreting the average number of specimen per lot, as depicted on Figure 6. With the exception of a few countries, the average number of specimen per lot is relatively low (average of 4.049 specimens per lot for the non-French specimens). Also, in the distribution of the type lots, France tends to be still over-represented in the dataset, though less markedly so (see Figure 7). This due to the fact that even though France is not a “hot spot” for myriapod biodiversity, it has been and still is an intensively studied area by the specialists working on the collection.

**Figure 8.**
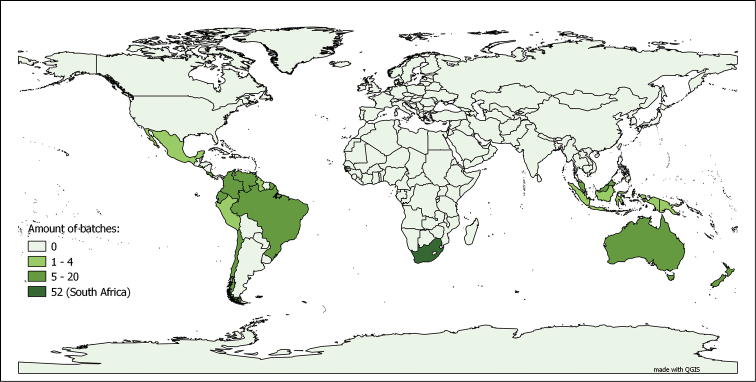
Origins of the onychophoran lots in the MNHN-MY dataset. Based on the 191 lots bearing information on country of origin, out of 202 lots in the dataset (entries up to 27.01.2015)

**Figure 9.**
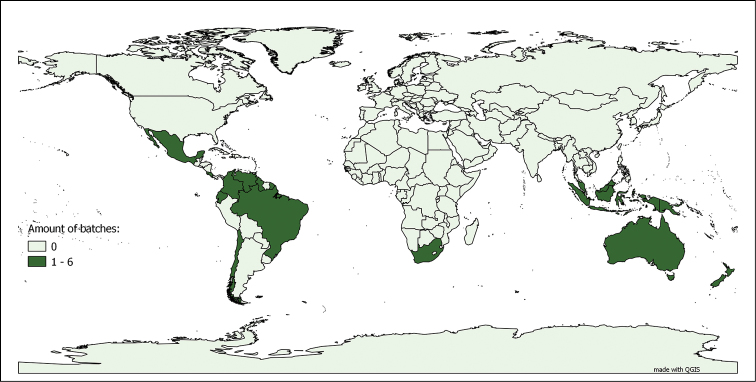
Origins of the onychophoran lots containing types in the MNHN-MY dataset. Based on the 56 lots of type specimens in the dataset, all of which bear information on country of origin (entries up to 27.01.2015)

**Onychophorans**: The onychophorans specimens come from the whole range of the group. One might be surprised to note in Figure 8 the importance of South Africa as a source of specimens in the collection. These specimens were mainly obtained through exchanges made between the MNHN and other institutes, such as the British Museum (Natural History). The distribution of type material in Figure 9 largely reflects the extensive descriptive work of Bouvier on this group.

**Temporal coverage**: 1833 – 2010.

**Figure 10.**
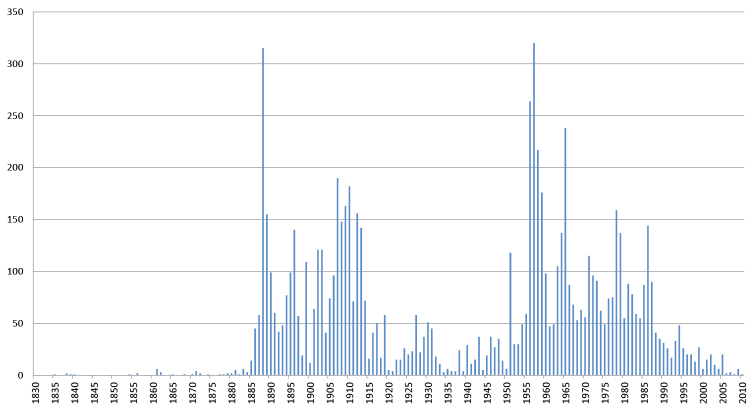
Number of myriapod lots by year of collection. Based on the 8 055 lots with collection date.

**Figure 11.**
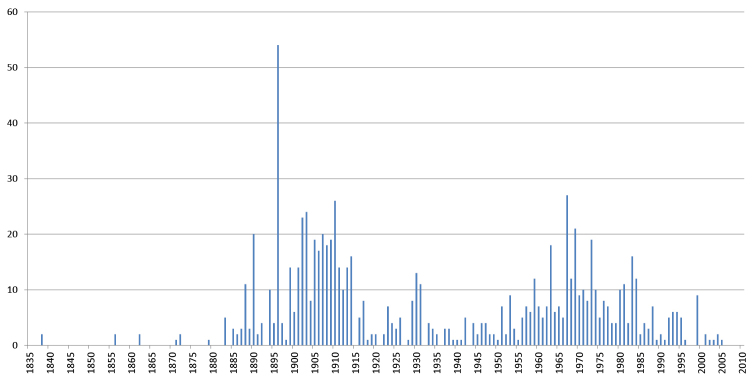
Number of myriapod lots containing type specimens by year of collection. Based on the 875 lots containing types with collection date.

**Myriapods**: The oldest myriapod specimen databased to date is MY4389, *Rhinocricus olivaceus* (Newport, 1844), which was collected in 1835 from Mexico. However, the acquisition rates were highest during two distinct periods, as can be seen in Figure 10. The first period corresponds to the activity of Brolemann from 1890 until his death in 1933. The second period, after World War II, corresponds to a renewal of interest in myriapodology at the MNHN, with the arrival of scientists such as Demange in 1942 and Mauriès in 1959. It should be noted, however, that specimens collected since 1990 are less well represented in the database than the older specimens, because they are still under study and will be progressively databased in the future. This is also illustrated in Figure 11. The number of type specimens per lot is higher during the first period than during Brolemann’s activity, mainly due to his intense collecting and important taxonomic work, including the description of a large number of new taxa.

**Figure 12.**
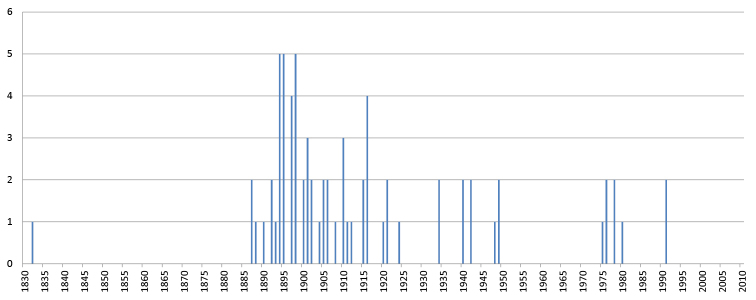
Number of onychophoran lots by year of collection. Based on the 72 lots with collection date.

**Onychophorans**: The oldest onychophoran specimen, is MY110 (formerly ON111), a type specimen of *Peripatus edwardsi* Blanchard, 1847, collected in 1833 from French Guyana. However, as can be seen in Figure 12, the major part of the collecting effort was carried out during Bouvier’s period of activity, from 1887 to 1931. Although Bouvier described a relatively large number of onychophoran taxa, of which many types are stored in this collection, only a few of these type specimens have a collection date.

## Natural collections description

**Parent collection identifier**: MNHN

**Collection name**: Myriapoda and Onychophora collection (MY) of the Muséum national d’Histoire naturelle (MNHN – Paris)

**Collection identifier**: MY

**Formation period**: 1985–2015

## Methods

**Specimen preservation method**: Alcohol and microscope slides

**Methods**: The specimens are gathered into lots, each consisting of one taxon from a single sampling event. The lots are mostly preserved in 75% alcohol in tubes plugged with cotton wool and placed in jars. Out of the databased collection, about 2% of the lots are in jars, rather than tubes, mainly due to their size. Over 97% of the jars contain one tube, 0.8% contain 2–5 tubes and less than 0.2% contain more than 5 tubes. These tubes are stored in glass jars filled with alcohol to avoid evaporation inside the tubes. In addition, preparations on microscope slides are also conserved, particularly for small specimens and dissected gonopods. Each databased lot receives an inventory number prefixed by the collection acronym (MY).

**Study extent description**: The collection is mainly used for studies on the systematics of myriapods and onychophorans. In terms of its size, composition and number of type specimens, it is a major resource for specialists of these taxa. Moreover, it has revealed an important number of unexpected new taxa within its previously less studied parts. The material from France and the Mediterranean areas, particularly Spain, have been extensively studied.

**Sampling description**: No single sampling protocol can be distinguished. Moreover, for most of the specimens, the sampling methodology is unknown, even if suspected to be by direct hand collecting in most cases.

## Datasets

### Dataset description

**Object name**: Darwin Core Archive Myriapoda and Onychophora collection (MY) of the Muséum national d’Histoire naturelle (MNHN – Paris).

**Character encoding**: UTF-8.

**Format name**: Darwin Core Archive format.

**Format version**: 1.0.

**Distribution**: http://collections.mnhn.fr/ipt/archive.do?r=mnhn-my and http://science.mnhn.fr/institution/mnhn/collection/my/item/search/form

**Publication date of data**: 24-03-2015.

**Language**: French.

**Metadata language**: English.

**Date of metadata creation**: 27-06-2014.

**Hierarchy level**: Dataset.
